# Harmonic scalpel versus flexible CO2 laser for tongue resection: A histopathological analysis of thermal damage in human cadavers

**DOI:** 10.1186/1477-7819-9-83

**Published:** 2011-08-01

**Authors:** Duncan F Hanby, Grayson Gremillion, Arthur W Zieske, Bridget Loehn, Richard Whitworth, Tamir Wolf, Anagha C Kakade, Rohan R Walvekar

**Affiliations:** 1 Department of Otolaryngology Head Neck Surgery, Louisiana State University Health Sciences Center, New Orleans, LA, USA; 2 Department of Pathology, Louisiana State University School of Medicine Health Sciences Center, New Orleans, LA, USA; 3 Department of Cell Biology and Anatomy, Louisiana State University Health Sciences Center, New Orleans, LA, USA; 4 Director of Research, Omni Guide LASER Systems, Cambridghe, MA, USA; 5 Statistician, Merial, New Jersey, USA

## Abstract

**Background:**

Monopolar cautery is the most commonly used surgical cutting and hemostatic tool for head and neck surgery. There are newer technologies that are being utilized with the goal of precise cutting, decreasing blood loss, reducing thermal damage, and allowing faster wound healing. Our study compares thermal damage caused by Harmonic scalpel and CO2 laser to cadaveric tongue.

**Methods:**

Two fresh human cadaver heads were enrolled for the study. Oral tongue was exposed and incisions were made in the tongue akin to a tongue tumor resection using the harmonic scalpel and flexible C02 laser fiber at various settings recommended for surgery. The margins of resection were sampled, labeled, and sent for pathological analysis to assess depth of thermal damage calculated in millimeters. The pathologist was blinded to the surgical tool used. Control tongue tissue was also sent for comparison as a baseline for comparison.

**Results:**

Three tongue samples were studied to assess depth of thermal damage by harmonic scalpel. The mean depth of thermal damage was 0.69 (range, 0.51 - 0.82). Five tongue samples were studied to assess depth of thermal damage by CO2 laser. The mean depth of thermal damage was 0.3 (range, 0.22 to 0.43). As expected, control samples showed 0 mm of thermal damage. There was a statistically significant difference between the depth of thermal injury to tongue resection margins by harmonic scalpel as compared to CO2 laser, (p = 0.003).

**Conclusion:**

In a cadaveric model, flexible CO2 laser fiber causes less depth of thermal damage when compared with harmonic scalpel at settings utilized in our study. However, the relevance of this information in terms of wound healing, hemostasis, safety, cost-effectiveness, and surgical outcomes needs to be further studied in clinical settings.

## Background

**Figure 1:**
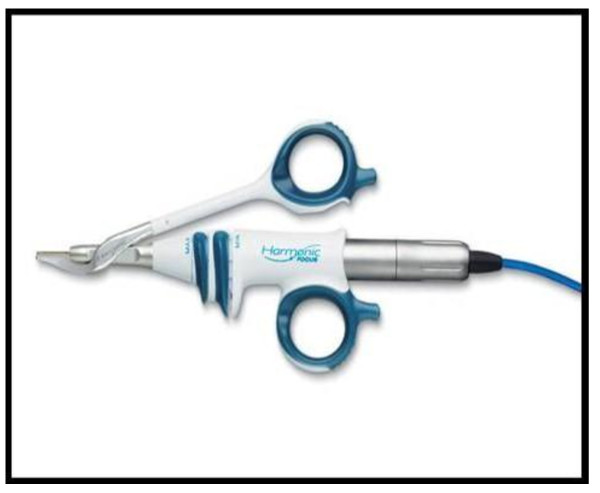
**Harmonic Focus**.

**Figure 2:**
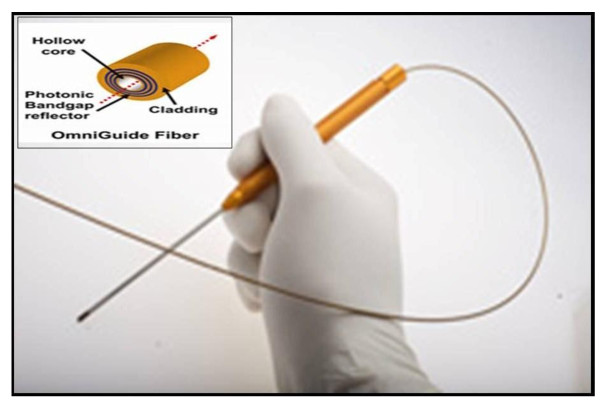
**OmniGuide Flexible Handheld CO2 Laser system with PBFA technology**.

There are multiple different options for a cutting tool in head and neck surgery. Monopolar cautery continues to be the gold standard and most commonly used cutting tool in most parts of the world. Monopolar cautery is extremely effective. However, it has been shown repeatedly to cause a significant amount of collateral tissue damage [1]. Thermal damage can have deleterious effects on wound healing, safety and clinical outcomes. Alternative technologies such as the harmonic scalpel (Figure 1) and carbon dioxide (CO2) laser are gaining popularity due to their similar effectiveness in cutting and coagulation with a lesser degree of collateral thermal damage. Multiple studies have demonstrated that the harmonic scalpel is a very effective and expedient tool for glossectomy [2]. The CO2 laser has also been proved to be an effective and precise cutting tool in the head and neck region [3-6]. Each modality has their advantages and disadvantages. The applicability of the laser particularly has been limited by line of sight in terms of its working capability. With the advent of the photonic band gap fiber assembly (PBFA), a flexible fiber CO2 delivery system developed by OmniGuide Inc, it is now possible to overcome these limitations, (Figure 2). The PBFA system allows the direct delivery of CO2 energy to regions in the head and neck where direct visualization is limited. This new technology has added versatility to the use of the laser and is being employed in all areas of otolaryngology with good surgical results. In our literature search we were unable to find studies that compare thermal damage between the new flexible CO2 laser fiber technology and the harmonic scalpel. Previous studies have demonstrated the superior tissue characteristics of these newer modalities compared with monopolar electrocautery, [6]. Therefore, our objective was to compare the tissue effects of the harmonic scalpel and PBFA carbon dioxide laser in tongue resections using a human cadaveric model.

## Methods

**Figure 3:**
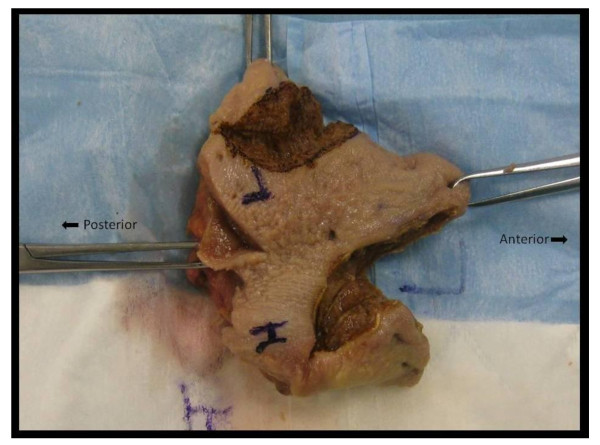
**Oral tongue specimen showing incisions using Harmonic scalpel and Flexible CO2 laser devices**. (H: Harmonic Scalpel; L: Flexible CO2 Laser)

Two fresh human cadaver heads were identified for the study. Surgical loupes were used for magnification. Oral tongue was exposed and incisions were made in the tongue akin to a tongue tumor resection using the harmonic scalpel and flexible C02 laser fiber at recommended settings of 5W for the harmonic scalpel and settings of 13W, 16W, and 18W for the PBFA carbon dioxide laser, (Neuro-L-Fiber LA090721AW-P2, Helium 85 PSI), (Figure 3). The margins of resection were sampled, labeled, fixed in formalin (10%), and sent for histological analysis to assess depth of thermal damage calculated in millimeters **(**Table 1**)**. The pathologist was blinded to the surgical tool used. Control tongue tissue was also sent for comparison as a baseline.

**Table 1:** Data values and Descriptive statistics

Thermal depth in mm	Method	Mean	Standard Deviation
0.75	harmonic scalpel	0.69	0.16
		
0.82	harmonic scalpel		
		
0.51	harmonic scalpel		
		
0.22	Co2 laser	0.30	0.08
		
0.24	Co2 laser		
		
0.43	Co2 laser		
		
0.31	Co2 laser		
		
0.28	Co2 laser		

### Data Entry and Statistical Analysis

A Microsoft Excel Spreadsheet and Statistical Package for the Social Science version 13.0 was maintained for the data entry and statistical analysis. Thermal depth between harmonic scalpel and CO2 laser was compared using Independent sample T-test. A p-value less than 0.05 was considered statistically significant.

## Results

**Figure 4:**
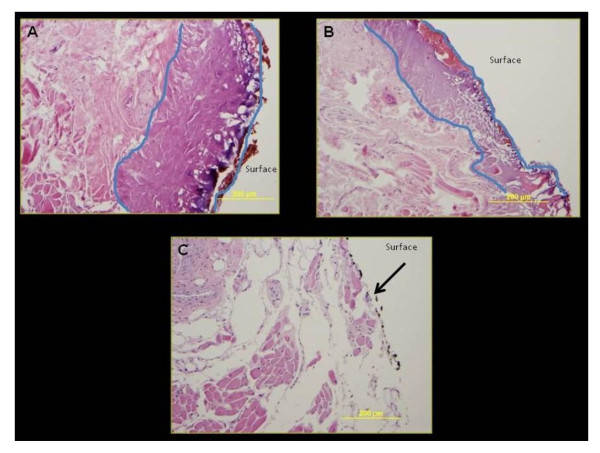
**H&E stained tongue specimens from Harmonic scalpel (A, 10× magnification), CO2 laser (B, 10× magnification), and control (C, 10× magnification)**. Regions enclosed in blue in Figures A and B, represent thermal damage. The black arrow in Figure C indicates black ink on the cut surface of the control specimen.

Three cadaveric tongue samples were analyzed for thermal damage with the harmonic scalpel. Five cadaveric tongue samples were analyzed for thermal damage with the PBFA carbon dioxide laser. The harmonic scalpel had a mean depth of thermal tissue damage of 0.69 mm, (0.51 - 0.82; SD 0.16). In comparison, the CO2 laser, applied in the same fashion had a mean depth of tissue damage of 0.30 mm, (0.22 - 0.43, SD 0.08), across power settings of 13W, 16W and 18W. The depth of thermal damage caused by the CO2 laser was significantly less than the harmonic scalpel, (p = 0.003) (Table 1, Figure 4A-C).

## Discussion

The CO2 laser was first introduced into Otolaryngology by Jako and Strong in 1972 [7]. At a wavelength of 10,600 nm, this laser is rapidly absorbed by the water in the tissues ensuring minimal thermal damage and spread. This makes the CO2 laser particularly well suited for use near critical anatomical structures [6]. Historically, the rapid absorption of this long wavelength laser by all known materials prevented its transmission via a flexible fiber. Therefore, most surgical CO2 lasers are applied via a bulky articulating arm either attached to a handpiece or to a micromanipulator mounted on an operating microscope. In this manner, the CO2 laser has been well established in the management of early glottic, supraglottic, oral and oropharyngeal and hypopharyngeal squamous cell carcinoma [7]. In areas where exposure is limited such as the posterior oropharynx, the bulky delivery system and the inability to use visualize the area being resected in the surgeon's line of sight have been factors that have limited a more wide spread use of this technology for this indication. Similarly, the laser can be an excellent tool for anterior oral cavity and anterior oral tongue resections. However, the bulk and cumbersome delivery system makes its use less attractive [3,7]. With the advent of the photonic band gap fiber assembly (PBFA), a flexible fiber CO2 delivery system developed by Omniguide Inc, it is now possible to overcome these limitations [6,7]. The PBFA system allows the direct delivery of CO2 energy to regions in the head and neck where direct visualization is limited. A variety of hand pieces allow laser energy to be provided along the plane of surgical dissection and in sync with the surgeon's line of sight. This facilitates precise surgery. In addition to increased maneuverability, a variable rate of gas is transmitted through the hollow core of the PBFA creating the added benefits of cooling the surgical site and clearing the field of debris, plume and blood [2,7].

The current limitations of the flexible CO2 laser fiber include a learning curve associated with its use in terms of maximizing its effectiveness. The PBFA also can be damaged if not used correctly. Although the tip of the fiber can provide tactile feedback to the surgeon, it is not robust enough to serve as a surgical dissector [1,2]. Another known limitation of the standard CO2 laser was that it was inefficient with respect to coagulation (vessels up to 1-2 mm in diameter). The new PBFA fiber can easily be focused to improve cutting and also defocused to coagulate by moving the tip of the laser fiber closer to the target or away from it^1^. However, a true assessment of the lasers utility and ease of use for coagulation can only be derived from clinical studies.

The harmonic scalpel is able to cut and coagulate at a lower temperature (max 150°C) using mechanical vibration at 55,500 cycles per second [3,8]. The harmonic scalpel like the laser causes less degree of thermal damage as compared to the monopolar cautery and has the ability to coagulate larger diameter vessels as compared to the laser ( 5 mm vs. 1-2 mm) which can be important in surgical resection of the tongue; an area that has a rich vascular supply [3,8].

As surgeons, we are all on a perpetual search for a perfect cutting tool. The ideal instrument would accomplish the necessary functions of cutting and coagulation while minimizing collateral tissue injury. Decreasing overall tissue injury has obvious clinical implications with regard to surgical precision and less obvious clinical implications with regard to outcomes measures like expediency in return to work and normal diet. The CO2 laser and the harmonic scalpel have proven to be superior to monopolar electrocautery in minimizing collateral tissue damage.

## Conclusion

In a cadaveric model, our study showed that flexible PFBA CO2 laser fiber causes less depth of thermal damage when compared with harmonic scalpel at recommended settings at the surgical margin. While it is tempting to extrapolate these findings into potential clinical benefits, further clinical studies are necessary to compare both surgical tools in terms of wound healing, hemostasis, safety, cost-effectiveness, and surgical outcomes.

## Conflict of interest

The authors declare that they have no competing interests.

## Authors' contributions

DFH: Helped in the write up of the paper and prepared the manuscript as First Author; GG: Helped in initial data collection, assisted in performing the study experiments, and literature review; AWZ: analyzed pathological slides and provided thermal depth results as pathologist on study; BL: literature review, editorial review and contributed to manuscript preparation and proofing; TW: provided insight into the capabilities of laser and participated in the study experiments as expert with OmniGuide Laser systems; ACK: statistics for the study; RRW: conceptualized the study, pooled resources to perform the study experiments, performed study experiments, preparation of manuscript, literature review, and editorial review, final editing and proofing prior to submission as Corresponding Author. All authors read and approved the final manuscript.
